# Paeoniflorin Ameliorates Fructose-Induced Insulin Resistance and Hepatic Steatosis by Activating LKB1/AMPK and AKT Pathways

**DOI:** 10.3390/nu10081024

**Published:** 2018-08-05

**Authors:** Yu-Cheng Li, Jing-Yi Qiao, Bao-Ying Wang, Ming Bai, Ji-Duo Shen, Yong-Xian Cheng

**Affiliations:** 1 Scientific Research and Experiment Center, Henan University of Chinese Medicine, Zhengzhou 450046, China; qiaojingyi618@126.com (J.-Y.Q.); wangbaoying812@163.com (B.-Y.W.); baiming666@126.com (M.B.); 2 College of Pharmacy, Henan University of Chinese Medicine, Zhengzhou 450046, China; lycdd1219@163.com; 3 Guangdong Key Laboratory for Genome Stability & Disease Prevention, School of Pharmaceutical Sciences, Shenzhen University Health Science Center, Shenzhen 518060, China; yxcheng@szu.edu.cn

**Keywords:** insulin resistance, hepatic steatosis, paeoniflorin, AMPK

## Abstract

The present study aimed to evaluate the effects of paeoniflorin on insulin resistance and hepatic steatosis induced by fructose. Male Sprague-Dawley rats were fed 20% fructose drink for eight weeks. The insulin sensitivity, serum lipid profiles, and hepatic lipids contents were measured. The results showed that paeoniflorin significantly decreased serum insulin and glucagon levels, improved insulin sensitivity and serum lipids profiles, and alleviated hepatic steatosis in fructose-fed rats. Moreover, paeoniflorin enhanced the phosphorylation level of AMP-activated protein kinase (AMPK) and protein kinase B (PKB/AKT) and inhibited the phosphorylation of acetyl coenzyme A carboxylase (ACC)1 in liver. Paeoniflorin also increased the hepatic carnitine palmitoyltransferase (CPT)-1 mRNA and protein expression and decreased the mRNA expression of sterol regulatory element-binding protein (SREBP)1c, stearyl coenzyme A decarboxylase (SCD)-1 and fatty acid synthetase (FAS). Furthermore, we found that paeoniflorin significantly increased the heptatic protein expression of tumor suppressor serine/threonine kinase (LKB)1 but not Ca^2+^/CaM-dependent protein kinase kinase (CaMKK)β. These results suggest that the protective effects of paeoniflorin might be involved in the activation of LKB1/AMPK and insulin signaling, which resulted in the inhibition of lipogenesis, as well as the activation of β-oxidation and glycogenesis, thus ameliorated the insulin resistance and hepatic steatosis. The present study may provide evidence for the beneficial effects of paeoniflorin in the treatment of insulin resistance and non-alcoholic fatty liver.

## 1. Introduction

Insulin resistance is a pathological condition that the insulin sensitive organ, such as liver and muscle, resist the action of insulin. To maintain normal blood glucose level, the pancreas β cell has to produce more amounts of insulin, which eventually may lead to the failure of islet β cell [1]. Insulin resistance is usually considered as the early stage of diabetes. Moreover, insulin resistance is highly linked with metabolic syndrome, non-alcoholic fatty liver (NAFLD), and cardiovascular disease [2,3]. In fact, hepatic steatosis impairs the hepatic insulin signaling and accelerates the development of insulin resistance. Insulin resistance will accelerate lipolysis and thereby aggravate fatty liver disease, which is now widely accepted as the “Two-hit” theory [4]. Therefore, it is critical to improve insulin resistance in treatment of NAFLD and metabolic syndrome.

In recent decades, increasing dietary fructose consumption is becoming a major risk factor contributing to the metabolic syndrome and NAFLD [5]. The overconsumption of fructose has been highly associated with insulin resistance, obesity, metabolic syndrome, and hepatic steatosis [4]. The restriction of fructose intake is beneficial for the improvement of NAFLD in patients [6]. Unlike glucose, fructose robustly accelerates the de novo lipogenesis in liver, promotes the production and secretion of triglyceride (TG) and very low–density lipoprotein (VLDL) [7]. Fructose impairs insulin signaling and thereby leads to hepatic and peripheral insulin resistance. The rodent model had been established by high-fructose diet and used for the study of pathophysiology and pharmacology of metabolic syndrome or NAFLD [8].

**Figure 1:**
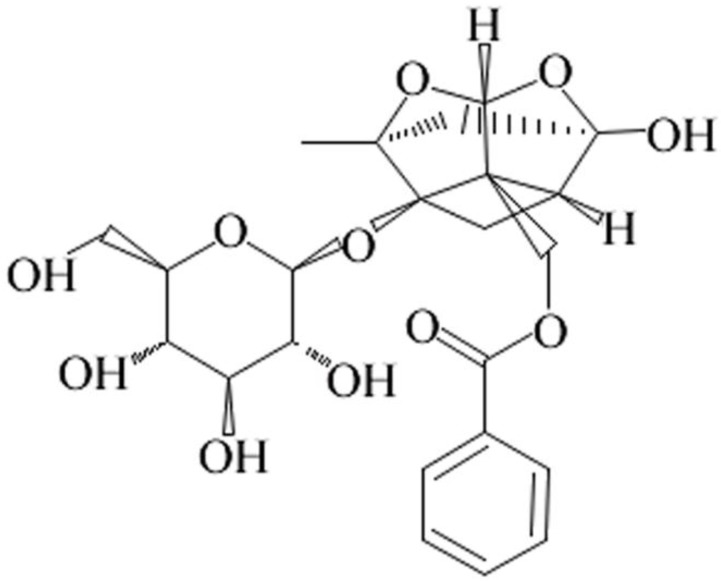
The chemical structure of paeoniflorin.

Paeoniflorin (Figure 1), a monoterpene glucoside component of *Paeonia lactiflora* Pall., possesses a variety of pharmacological activities, including antioxidant, hepatoprotection, hypolipidemic, and hypoglycemic properties. Recent studies reported that paeoniflorin can ameliorate high-fat diet–induced steatohepatitis by enhancing insulin signaling, promoting fatty acid oxidation, and inhibiting lipids synthesis in liver [9,10,11]. However, it still remains unclear whether paeoniflorin could improve fructose induced insulin resistance and hepatic steatosis. The present study was designed to investigate the effects of paeoniflorin on fructose-induced insulin resistance and hepatic steatosis and explore the underlying mechanism by which paeoniflorin protects against hepatic steatosis and insulin resistance in fructose-fed rats.

## 2. Materials and Methods

### 2.1. Materials

Paeoniflorin (98% of HPLC grade purity) was purchased from Aladdin (Shanghai, China). d-fructose was supplied by Xiwang Food Co., Ltd. (Jinan, China). Pioglitazone hydrochloride was obtained from Deyuan Pharmaceutical Co., Ltd. (Lianyungang, China). The assay kits of glycogen, non-esterified fatty acids (NEFA), and TG were purchased from Nanjing Jiancheng Bionengineering Institute (Nanjing, China). Insulin and glucagon ELISA kits were purchased from CUSABIO Biotechnology Company (Wuhan, China). Primary antibodies of LKB1 (#3050), AMPKα (#5832), p-AMPKα (Th172, #2535), AKT (#9272), p-AKT (Ser473, #9271), ACC1 (#3662), and p-ACC1 (Ser79, #3661) were from Cell Signaling Technology (Danvers, MA, USA). Antibody of CPT1a (15184-1-AP) and CaMKKβ (11549-1-AP) was from Proteintech (Wuhan, China). Antibody of GAPDH and HRP-conjugated goat anti rabbit IgG were from Kangcheng (Shanghai, China).

### 2.2. Animals

Male Sprague-Dawley rats weighing 180~200 g were obtained from Hunan Slac Animal Center (Changsha, China). Animals were acclimatized one week before the experiment beginning. All rats were individually housed in a temperature-controlled room (23–25 °C) with a 12-h light/dark cycle and began at 20:00. Subsequently, the rats were randomly divided into six groups (*n* = 8): Control group (Saline), fructose group (20% Fructose drink), PF-10 group (20% Fructose drink and 10 mg/kg paeoniflorin), PF-20 group (20% Fructose drink and 20 mg/kg paeoniflorin), PF-40 group (20% Fructose drink and 40 mg/kg paeoniflorin), pioglitazone group (20% Fructose drink and 10 mg/kg pioglitazone). As an insulin sensitizer, pioglitazone is widely used for treating insulin resistance. We choose pioglitazone as a positive control drug mainly based on previous study and other reports [12,13,14]. Paeoniflorin and pioglitazone were suspended in saline and given by oral gavage at 4 mL/kg for eight weeks. Control and Fructose group received equal volume of saline. All experimental protocols were approved by the Henan University of Chinese Medicine Animal Ethics Committee (DWLL20130025).

### 2.3. Oral Glucose Tolerance Test

The oral glucose tolerance test (OGTT) was performed at the Thursday in week 8 (day 53). Followed a 14-h fasting, all rats were orally administrated with 50% glucose solution (1.5 g/kg), and the tail vein blood were collected at 0, 30, 60, 90, 120 min after administration. The blood glucose was assayed by an ACCU-CHEK blood glucose meter (Roche Diagnostics Ltd. Company, Shanghai, China).

### 2.4. Blood Biochemical Analysis

To avoid the potential interference of OGTT on hormone levels, the rats were allowed three days for recovery. At day 56, the fasting blood were collected by orbital venous between 9:00 and 11:00. The serum was separated by centrifuged at 4 °C, 4000 rpm for 10 min. The rats were killed by decapitation. The liver tissue was divided into small pieces (100 mg) and immediately frozen in liquid nitrogen and then stored at −80 °C until analysis. A piece of liver tissue was fixed in 10% formalin for preparation of paraffin slice. Another piece of liver tissue was prepared to frozen slice for oil-red O staining. The serum levels of glucose, TG, total cholesterol (TC), high-density lipoprotein cholesterol (HDL-C), low-density lipoprotein cholesterol (LDL-C), albumin (ALB), aspartate aminotransferase (AST) and alanine aminotransferase (ALT) were determined by AU400 automatic biochemical analyzer (OLYMPUS Co., Ltd, Tokyo, Japan). The serum levels of insulin and glucagon were measured by ELISA kits. The homeostasis model assessment insulin resistance index (HOMA-IR) was concluded as the formula: HOMA-IR = Fasting glucose (mmol/L) × Fasting insulin (mIU/L)/22.5. The hepatic glycogen contents were determined according to the instructions of manufacturer.

### 2.5. Hepatic Lipids Analyses

For lipid analyses, the liver lipids were extracted according to the method of Folch [15] with slight modification. In brief, the liver tissue was weighted and homogenized with a 20-fold volume of chloroform/methanol (2:1) mixture. After shaking for 15–20 min, the deposition was removed by centrifuge at 2000× *g* for 10 min. Then, a 0.2-fold volume of water was added, and the mixture was centrifuged at 2000× *g* for 10 min. The lower layer (chloroform phase) was transferred to a new tube. After drying by nitrogen, the weight of lipids was accurately weighted.

### 2.6. Histological Analysis

For Oil-red O staining, the liver tissues were snap-frozen at −58 °C. A 6-μm-thick cryostat sections were prepared and stained with Oil-red O for 5–10 min. After washed with 60% isopropyl alcohol, the sections were re-stained by hematoxylin. The area of staining was counted by Image-Pro Plus 6.0 software (Media Cybernetics, Silver Spring, MD, USA). For hematoxylin and eosin (HE) staining, the liver tissue was fixed in 10% formalin for 36 h, the paraffin-embedded specimens were sections at 5 μm and then stained with hematoxylin-eosin reagents.

### 2.7. Quantitative Real-Time polymerase chain reaction (qRT-PCR)

Total RNA was extracted from liver using Trizol reagent following the manufacturer’s protocol (Invitrogen, Carlsbad, CA, USA). First strand cDNA was synthesized using M-MLV first chain synthesis kit (Invitrogen, Carlsbad, CA, USA). qPCR was performed using a SYBR green PCR kit (Thermo Fisher Scientific Inc., Waltham, MA, USA) in ABI step one plus system (Applied Biosystems, Foster City, CA, USA). The amplification process time was as follows: 95 °C for 2 min; 40 cycles at 95 °C for 20 s, 60 °C for 15 s, and 72 °C for 30 s. The specific primers were synthesized by Sangon Biotech Co., Ltd. (Shanghai, China), and the sequences are presented in Table 1. GAPDH was used as internal reference gene to normalize gene expression.

### 2.8. Western Blotting

The liver tissues were homogenized in 10 *wt*/vol RIPA buffer (50 mM Tris, pH 7.4, 150 mM NaCl, 1% Triton X-100, 1% sodium deoxycholate, 0.1% SDS, 1 mM EDTA) with 0.1 mM PMSF and phosphatase inhibitor cocktail (CWBIO, Beijing, China). The homogenate was placed on ice for 20 min. After centrifugation at 12,000× *g* for 20 min at 4 °C, the supernatants were collected, and the protein concentration was determined using a BCA kit (CWBIO, Beijing, China). Equal proteins (50 μg) were separated by 10% SDS-PAGE and transferred to PVDF membranes (Millipore, Shanghai, China). After 2 h blocking by 5% BSA, the membranes were incubated with primary antibodies (AMPK, 1:1000; p-AMPK, 1:500; AKT, 1:1000; p-AKT, 1:1000; ACC1, 1:1000; p-ACC1, 1:500; CPT1a, 1:500; LKB1, 1:1000; CaMKK, 1:500) overnight at 4 °C. After three washes with TBST, the blotted membranes were incubated with HRP-conjugated secondary antibody for 1 h at room temperature. The immunoreactive bands were visualized using ECL reagent (Merck KGaA, Darmstadt, Germany) and quantified by IPP6.0.

### 2.9. Statistical Analysis

All data are expressed as Mean ± SEM. The data are analyzed by one-way ANOVA followed by Dunnett’s *post hoc* test. A value of *p* < 0.05 is considered as statistically significance.

## 3. Results

### 3.1. Paeoniflorin Reversed the Metabolic Abnormalities Induced by Fructose

**Figure 2:**
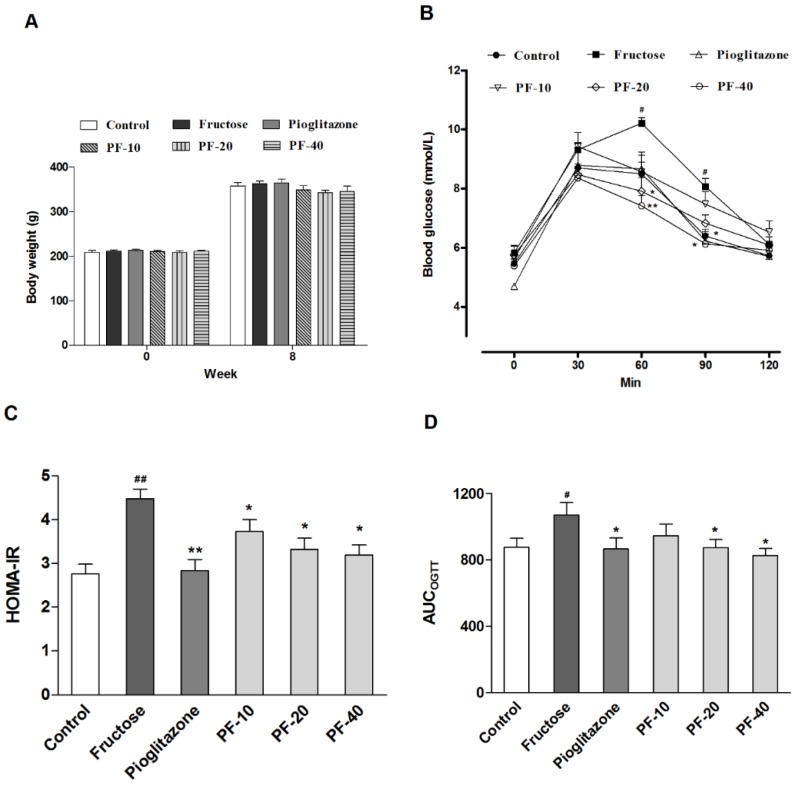
Effects of paeoniflorin on the body weight and insulin sensitivity in rats. Data are expressed as the mean ± SEM (*n =* 8). (**A**) Body weight. (**B**) The oral glucose tolerance test. (**C**) The homeostasis model assessment insulin resistance index (HOMA-IR) index. (**D**) The area under curve during oral glucose tolerance test (OGTT). Data was analyzed by one-way ANOVA followed by Dunnett’s post hoc test. ^#^ *p* < 0.05 and ^##^ *p* < 0.01 vs. control group. * *p* < 0.05 and ** *p* < 0.01 vs. fructose group. PF-10: 10 mg/kg of paeoniflorin; PF-20: 20 mg/kg of paeoniflorin; PF-40: 40 mg/kg of paeoniflorin. HOMA-IR: Homeostasis model assessment insulin resistance index; OGTT: Oral glucose tolerance test; AUC: Area under curve.

Although fructose did not increase the blood glucose level, the fructose-fed rats exhibited a significant elevated serum insulin (*p* < 0.001) and glucagon levels (*p* < 0.001). Insulin resistance was also observed in OGTT. Paeoniflorin enhanced the insulin sensitivity (*p* < 0.05), decreased serum insulin (10 mg/kg: *p* < 0.05; 20 mg/kg: *p* < 0.01; 40 mg/kg: *p* < 0.01) and glucagon levels (20 mg/kg: *p* < 0.01; 40 mg/kg: *p* < 0.001) in fructose-fed rats. In addition, paeoniflorin significantly restored the elevation of serum TG (*p* < 0.01), TC (20 mg/kg: *p* < 0.05; 40 mg/kg: *p* < 0.01), HDL-C (*p* < 0.05), LDL-C (*p* < 0.01) and NEFA (10 mg/kg: *p* < 0.01; 20 mg/kg: *p* < 0.001; 40 mg/kg: *p* < 0.001). Pioglitazone also decreased the serum levels of TG (*p* < 0.01), TC (*p* < 0.05), HDL-C (*p* < 0.05), LDL-C (*p* < 0.01) and NEFA (*p* < 0.001). Pioglitazone significantly decreased serum insulin (*p* < 0.001) and glucagon (*p* < 0.05) levels, and improved insulin sensitivity (*p* < 0.05) in fructose-fed rats. The body weight had no significant alteration among groups. The results were shown in Table 2 and Figure 2.

### 3.2. Paeoniflorin Improved the Liver Function in Fructose Rats

To investigate the effects of paeoniflorin on the liver function in fructose-fed rats, we examined the serum levels of ALB, AST, and ALT. As shown in Table 2, there were no significant changes on these indexes between fructose and control groups. Paeoniflorin significantly decreased the AST level (10 mg/kg: *p* < 0.05; 20 mg/kg: *p* < 0.01; 40 mg/kg: *p* < 0.001). Pioglitazone had no effect on liver function in fructose-fed rats.

### 3.3. Paeoniflorin Reversed Fructose-Induced Hepatic Lipids Accumulation and the Reduction of Hepatic Glycogen

**Figure 3:**
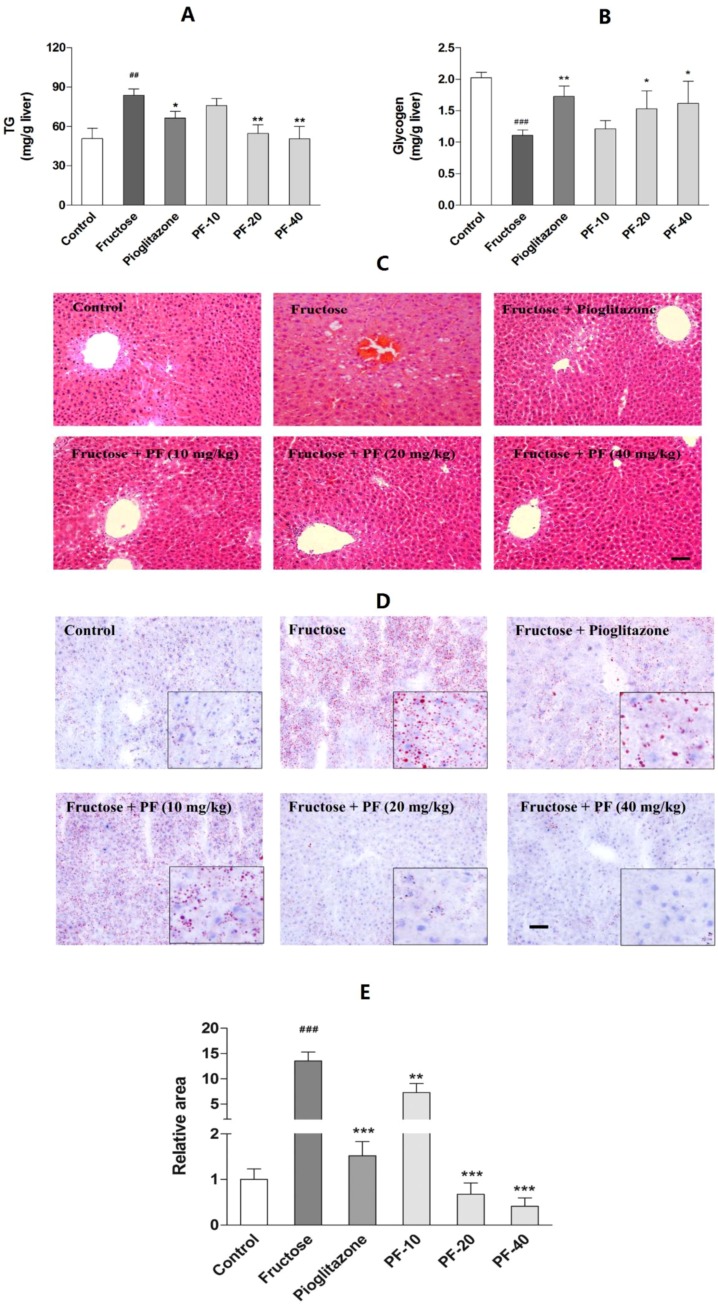
Effects of paeoniflorin on the hepatic lipids and glycogen contents. (**A**) Hepatic lipids contents. (**B**) Hepatic glycogen contents. (**C**) HE staining (200 ×). (**D**) Oil-red O staining (200 ×). Insets from the images are magnified five times in order to highlight the lipid-staining morphology. (**E**) Relative area of Oil-red O staining. Data were expressed as the mean ± SEM. (*n =* 8). Data was analyzed by one-way ANOVA followed by Dunnett’s *post hoc* test. ^##^ *p* < 0.01 and ^###^ *p* < 0.001 vs. control group. * *p* < 0.05, ** *p* < 0.01 and *** *p* < 0.001 vs. fructose group. PF-10: 10 mg/kg of paeoniflorin; PF-20: 20 mg/kg of paeoniflorin; PF-40: 40 mg/kg of paeoniflorin. The scale bar represents a length of 50 μm.

To examine the hepatic lipids contents, we exacted the hepatic total lipids. Fructose significantly increased the hepatic lipids contents (*p* < 0.01). Histological results also displayed a slight steatosis in fructose group. Paeoniflorin (20 mg/kg: *p* < 0.01; 40 mg/kg: *p* < 0.01) and pioglitazone (*p* < 0.05) reduced lipids accumulation. In addition, paeoniflorin (20 mg/kg: *p* < 0.05; 40 mg/kg: *p* < 0.05) and pioglitazone (*p* < 0.01) significantly reversed the reduction of hepatic glycogen in fructose-fed rats. The data are shown in Figure 3.

### 3.4. Paeoniflorin Inhibited the Hepatic Lipogenesis and Promoted Fatty Acid Oxidation

**Figure 4:**
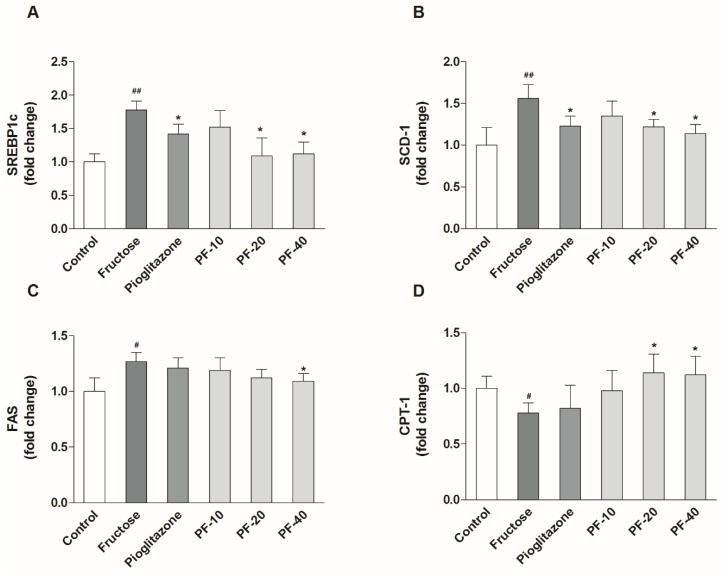
Effects of paeoniflorin on the mRNA expression of SREBP1c, SCD-1, FAS and CPT-1 in liver. (**A**) The mRNA expression of SREBP1c. (**B**) The mRNA expression of SCD-1. (**C**) The mRNA expression of FAS. (**D**) The mRNA expression of CPT-1. Data were normalized by the abundance of GAPDH and expressed as the relative value to control. Data were expressed as the mean ± S.E.M. (*n =* 6) and analyzed by one-way ANOVA followed by Dunnett’s *post hoc* test. ^#^ *p* < 0.05 and ^##^ *p* < 0.01 vs. control group. * *p* < 0.05 vs. fructose group. PF-10: 10 mg/kg of paeoniflorin; PF-20: 20 mg/kg of paeoniflorin; PF-40: 40 mg/kg of paeoniflorin. SREBP1c: Sterol regulatory element-binding protein 1c; SCD-1: Stearyl coenzyme A decarboxylase; FAS: Fatty acid synthetase; CPT-1: Carnitine palmitoyltransferase I.

Fructose significantly increased the mRNA expression of SREBP1c (*p* < 0.01), SCD-1 (*p* < 0.01), FAS (*p* < 0.05) and decreased CPT-1 (*p* < 0.05) expression in liver. 20 mg/kg of paeoniflorin reversed the changes of SREBP1c (*p* < 0.05), SCD-1 (*p* < 0.05) and CPT-1 (*p* < 0.05), and 40 mg/kg of paeoniflorin succeed in restoring all the abnormal expression of these genes (*p* < 0.05) in fructose-fed rats. Pioglitazone only reversed hepatic expression of SREBP1c (*p* < 0.05) and SCD-1 (*p* < 0.05) in fructose-fed rats. The data were shown in Figure 4.

### 3.5. Paeoniflorin Enhanced the Activities of LKB1/AMPK and AKT Signaling in Liver

**Figure 5:**
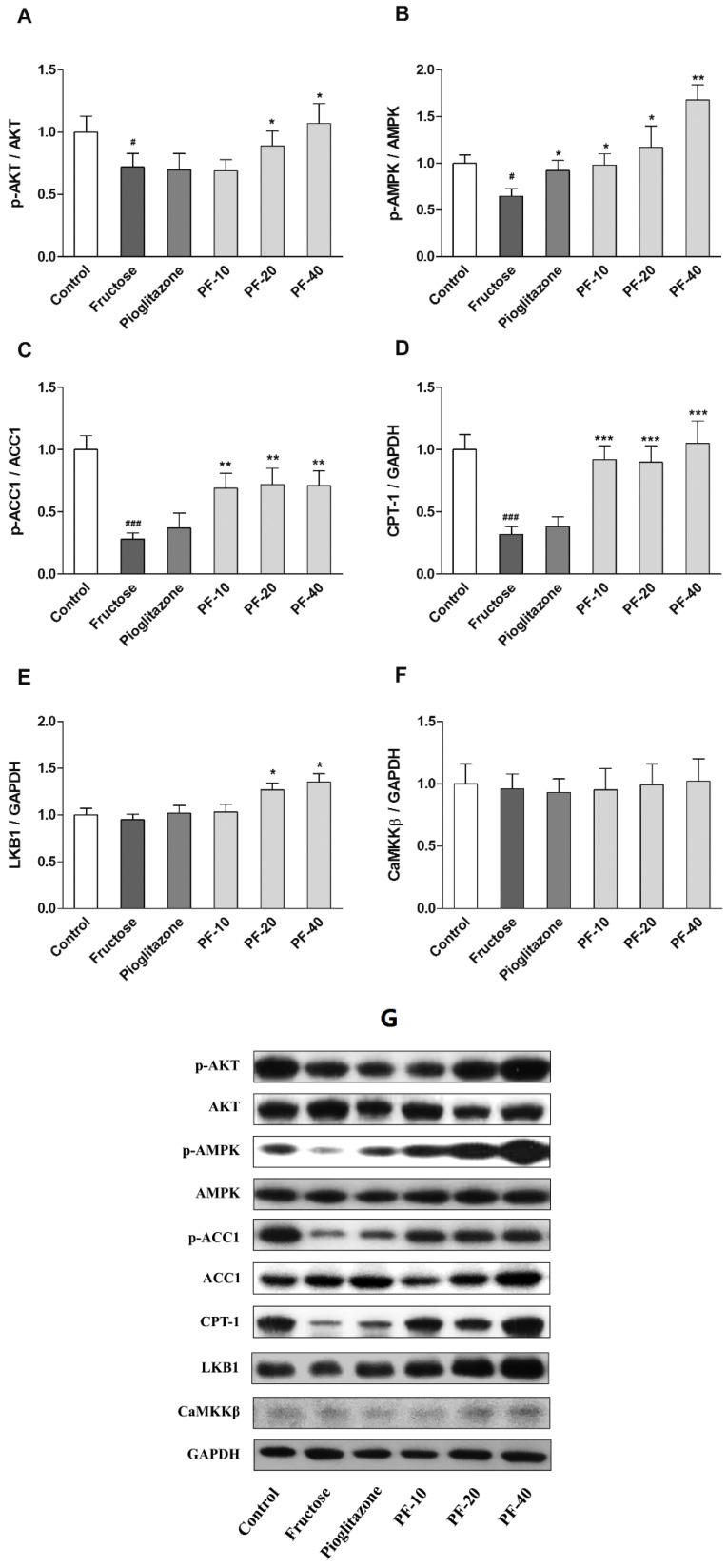
Effects of paeoniflorin on the body weight and insulin sensitivity in rats. (**A**) The phosphorylation level of AKT. (**B**) The phosphorylation level of AMP-activated protein kinase (AMPK). (**C**) The phosphorylation level of ACC1. (**D**) The protein expression of CPT-1. (**E**) The protein expression of LKB1. (**F**) The protein expression of CaMKKβ. (**G**) Immunoblot bands. Data were normalized by the abundance of GAPDH and expressed as the relative value to control. Data were expressed as the mean ± SEM (*n =* 6) and analyzed by one-way ANOVA followed by Dunnett’s *post hoc* test. ^#^ *p* < 0.05 and ^###^ *p* < 0.001 vs. control group. * *p* < 0.05, ** *p* < 0.01 and *** *p* < 0.001 vs. fructose group. PF-10: 10 mg/kg of paeoniflorin; PF-20: 20 mg/kg of paeoniflorin; PF-40: 40 mg/kg of paeoniflorin; AKT: Protein kinase B; AMPK: AMP-activated protein kinase; ACC1: Acetyl coenzyme A carboxylase; CPT-1: Carnitine palmitoyltransferase I; GAPDH: Glyceraldehyde-3-phosphate dehydrogenase; LKB1: Tumor suppressor serine/threonine kinase 1; CaMKKβ: Ca^2+^/CaM-dependent protein kinase kinase β.

Fructose significantly decreased the phosphorylation of AMPK (Thr 172) and AKT (Ser 439) (*p* < 0.05). Paeoniflorin significantly increased the phosphorylation levels of AMPK and AKT in a dose dependent manner (*p* < 0.05). The CPT1a expression and phosphorylation level of ACC1 (Ser 79) were significantly decreased in fructose group (*p* < 0.001). Paeoniflorin increased the CPT1a expression (*p* < 0.001) and the phosphorylation level of ACC1 (*p* < 0.01). Moreover, paeoniflorin (20 and 40 mg/kg) significantly increased LKB1 expression (*p* < 0.05) but did not affect CaMKKβ. Pioglitazone only increased the phosphorylation of AMPK in fructose-fed rats (*p* < 0.05). The data were shown in Figure 5.

## 4. Discussion

The main objective of the present study was to explore the underlying mechanisms by which paeoniflorin attenuates insulin resistance and hepatic steatosis induced by fructose in rats. Our results demonstrated the protective effects of paeoniflorin against fructose and suggested the mechanism might be mediated by activating LKB1/AMPK and AKT signaling pathways.

We first demonstrated that paeoniflorin was able to reverse metabolic abnormalities induced by fructose. Paeoniflorin significantly enhanced insulin sensitivity, improved serum lipid profiles and reduced the fasting serum insulin and glucagon level in fructose-fed rats. A previous study reported that fructose increased AST levels while did not affect ALT levels [16]. However, there was no significant difference in liver function between fructose and control group in the present study. The difference could be attributed to the shorter period of fructose drinking in our study (eight weeks vs. 12 weeks). However, paeoniflorin significantly reduced the AST levels when compared with fructose group.

Previous studies reported that the rats fed a high-fat diet for three days developed hepatic insulin resistance prior to peripheral insulin resistance and suggested the hepatic TG accumulation increased the susceptibility to insulin resistance or glucose intolerance [17]. Hepatic lipids accumulation mainly results from the increased hepatic de novo lipogenesis, dietary fat or influx of blood free fatty acids. A high-fat diet caused fatty liver due to over intake of dietary fatty acid. Fructose strongly promotes de novo lipogenesis in liver and worsens insulin resistance [7,18]. Insulin plays a key role in preventing lipolysis of adipose tissues. Insulin resistance will accelerate lipolysis and increase blood free fatty acids inflow to the liver. A previous study revealed that the TG synthesized via de novo lipogenesis was significantly enhanced in NAFLD subjects compared with normal [19]. As a monosaccharide, fructose is mainly absorbed via glucose transporter (GLUT) 2 and phosphorylated to fructose 1-phosphate (F1P) by fructokinase in hepatocyte. Although a recent report showed the dietary fructose is primarily cleared by the small intestine, high doses of fructose overwhelm the absorptive capacity of small intestine and resulting in fructose reaching the liver [20]. Fructose-1-phophate can be metabolized to glyceraldehyde-3 phosphate and dihydroxyacetone phosphate (DHAP) and further converted to the substrate for fatty acid synthesis in the regulation of ACC1, FAS and SCD-1. A tracer study also displayed that fructose is acutely incorporated into glycerol and FFA [21]. SREBP1c is a key transcription factor by binding to its target genes, such as FAS, ACC, and SCD-1, and thereby initiates hepatic lipogenesis [22]. Previous studies showed that fructose strongly accelerated lipogenesis by upregulating the expression of SREBP1c, FAS, SCD-1, and ACC1 [4,23,24,25]. In line with previous studies, we also observed a significant increased mRNA expression of SREBP1c, FAS, and SCD-1. We found that paeoniflorin significantly down-regulated the mRNA expression of SREBP1c, FAS, and SCD-1, accompanied by the decrease in hepatic lipids content. These results are similar to other reports in NAFLD mice or rats induced by high-fat diet [9,11]. The reduced steatosis is beneficial for improving insulin sensitivity.

AMPK is a serine/threonine protein kinase that serves as an energy sensor in regulation of cellular metabolism. AMPK is activated by the raising AMP/ATP ratio or other upstream kinase. Activated AMPK accelerated the fatty acid β-oxidation and TCA to generate ATP, as well as inhibit the fatty acid synthesis and gluconeogenesis to reduce ATP consumption. Several AMPK agonists, such as berberine and metformin, were well known to improve insulin resistance and alleviate NAFLD [26,27]. Therefore, AMPK is regarded as a key target in treatment of insulin resistance, type 2 diabetes, and NAFLD [28,29]. We found that paeoniflorin upregulated the phosphorylation level of AMPK at Thr172. Phosphate-AMPK inhibited fatty acid synthesis via promoting phosphorylation and inactivation of ACC1. ACC1 can catalyze acyl-CoA to malonyl-CoA and thus provide the substrate for de novo lipogenesis. To avoid the futile cycle of fatty acid synthesis and β-oxidation, malonyl-CoA acts as an allosteric inhibitor to inhibit CPT-1, a rate limiting enzyme of fatty acid β-oxidation [30]. Therefore, the inactivation of ACC1 removes the inhibitory effect of malonyl-CoA, and thereby indirectly activated CPT-1 to accelerate fatty acid β-oxidation. AMPK also inhibited the translocation of SREBP1c into nuclei [31], thus downregulated the expression of lipogenesis related genes. Recent studies showed that fructose promoted the production of methylglyoxal, a precursor or product of TG. Methylglyoxal can inhibit the activation of AMPK through binding to the arginine residues of AMPKγ subunit and interfering the allosteric activation effect of AMP [32]. It is well known that the activation of AMPK requires phosphorylation on Thr 172 at catalytic α-subunit by upstream kinases, LKB1, and CaMKKβ [33,34]. We found that paeoniflorin significantly increased the expression of LKB1 without affecting CaMKKβ. The present study verified that paeoniflorin activate LKB1/AMPK pathway, thereby increase CPT1a expression and inactivate ACC1 by phosphorylation, which further promoting β-oxidation and inhibiting lipogenesis.

AKT is a key kinase of insulin signaling, response for lots of downstream effects of insulin, such as promoting glucose transport and glycogen synthesis. Previous studies showed that fructose significantly decreased the phosphorylation level of AKT and inhibited glycogen synthesis and glucose transport [35]. These alterations can be considered as a feature of hepatic insulin resistance. We found that the hepatic glycogen contents were significantly reduced in fructose rats, which was reversed by paeoniflorin treatment. Increased glycogen storage could be attributed to the upregulated phosphorylation level of AKT (Ser 473) induced by paeoniflorin, which further verified the improvement of insulin signaling. These results are consistent with previous reports [10,36]. It is noticeable that the activation of AMPK will inhibit glycogenesis, whereas AKT promotes it. This paradox could be explained as that AMPK inhibit glycogenesis due to the lack of ATP. However, the improvement of insulin signaling, the elevated β-oxidation level, as well as the decrease of glucagon all contribute to the synthesis of glycogen.

## 5. Conclusions

In conclusion, the present study demonstrated that paeoniflorin reversed fructose-induced insulin resistance and hepatic steatosis, suggesting the mechanism might be involved in the activation of LKB1/AMPK and insulin signaling, which resulted in the inhibition of lipogenesis and the activation of β-oxidation and glycogenesis. These results might provide evidence for the beneficial effects of paeoniflorin in treatment of insulin resistance and NAFLD.

## Figures and Tables

**Table 1:** The sequences of primers for qRT-PCR.

Genes	Accession	Sense	Antisense
SREBP1c	NM_001276707	5′-CCATGGACGAGCTACCCTTC-3′	5′-GCCTGTGTCTCCTGTCTCAC-3′
SCD-1	AF509569.1	5′-CCTGGCTTACGACCGGAAA-3′	5′-CAGGAACTCAGAAGCCCAG-3′
FAS	NM_012820.1	5′-TGTGGGGTGGAAATCATCGG-3′	5′-CATTGCTCCTTTGGGGTTGC-3′
CPT-1	NM_064320.3	5′-ACGAGCCGATTGGGCTAAA-3′	5′-ACCAACGATCGTGAGCCTTT-3′
GAPDH	NM_017008.4	5′-AGTGCCAGCCTCGTCTCATA-3′	5′-GGTAACCAGGCGTCCGATA-3′

SREBP1c: Sterol regulatory element-binding protein 1c; SCD-1: Stearyl coenzyme A decarboxylase; FAS: Fatty acid synthetase; CPT-1: Carnitine palmitoyltransferase I; GAPDH: Glyceraldehyde-3-phosphate dehydrogenase.

**Table 2:** Effects of paeoniflorin on the serum biochemical indicators in fructose-fed rats.

	Control	Fructose	Pioglitazone	PF-10	PF-20	PF-40
TG (mmol/L)	0.87 ± 0.05	1.14 ± 0.10 ^#^	0.80 ± 0.06 **	0.96 ± 0.09	0.85 ± 0.08 **	0.71 ± 0.09 **
TC (mmol/L)	1.92 ± 0.10	2.37 ± 0.08 ^##^	2.05 ± 0.05 *	2.16 ± 0.12	2.05 ± 0.06 *	1.97 ± 0.07 **
HDL-C (mmol/L)	0.63 ± 0.06	0.76 ± 0.05 ^#^	0.57 ± 0.03 *	0.69 ± 0.07	0.60 ± 0.05 *	0.62 ± 0.04 *
LDL-C (mmol/L)	0.95 ± 0.07	1.59 ± 0.10 ^###^	1.20 ± 0.05 **	1.37 ± 0.09 *	1.23 ± 0.04 **	1.18 ± 0.08 **
NEFA (mmol/L)	0.92 ± 0.11	1.53 ± 0.07 ^###^	0.88 ± 0.07 ***	1.15 ± 0.08 **	1.07 ± 0.07 ***	0.92 ± 0.05 ***
Insulin (mIU/L)	11.7 ± 0.5	18.3 ± 0.5 ^###^	12.6 ± 0.8 ***	15.9 ± 0.3 *	14.1 ± 0.9 **	14.5 ± 0.9 **
Glucagon (pg/mL)	194.0 ± 4.6	245.6 ± 8.6 ^###^	227.7 ± 7.0 *	235.4 ± 10.8	206.8 ± 9.4 **	188.7 ± 9.5 ***
Glucose (mmol/L)	5.27 ± 0.09	5.43 ± 0.13	5.11 ± 0.16	5.19 ± 0.16	5.08 ± 0.13	4.98 ± 0.25
ALB (g/L)	37.9 ± 2.9	37.2 ± 1.4	37.4 ± 3.5	37.4 ± 4.3	38.9 ± 2.9	38.5 ± 1.9
AST (IU/L)	17.1 ± 0.7	18.8 ± 0.4	16.9 ± 0.6	15.3 ± 0.8 *	12.8 ± 0.5 **	12.2 ± 0.9 **
ALT (IU/L)	7.70 ± 1.34	8.23 ± 0.75	9.42 ± 1.54	9.34 ± 0.82	8.23 ± 1.15	8.84 ± 0.95

Data are expressed as the mean ± SEM. (*n =* 8). Data are analyzed by one-way ANOVA followed by Dunnett’s *post hoc* test. ^#^ *p* < 0.05, ^##^ *p* < 0.01 and ^###^ *p* < 0.001 vs. control group. * *p* < 0.05, ** *p* < 0.01 and *** *p* < 0.001 vs. fructose group. PF-10: 10 mg/kg of paeoniflorin; PF-20: 20 mg/kg of paeoniflorin; PF-40: 40 mg/kg of paeoniflorin. TG: Triglyceride; TC: Total cholesterol; HDL-C: High density lipoprotein cholesterol; LDL-C: Low density lipoprotein cholesterol; NEFA: Non-esterified fatty acid; ALB: Albumin; AST: Aspartate aminotransferase; ALT: Alanine aminotransferase.

## References

[1] Gisela W. (2005). Insulin and Insulin Resistance. Clin. Biochem. Rev..

[2] Reaven G.M. (1988). Banting lecture 1988. Role of insulin resistance in human disease. Diabetes.

[3] Asrih M., Jornayvaz F.R. (2015). Metabolic syndrome and nonalcoholic fatty liver disease: Is insulin resistance the link?. Mol. Cell. Endocrinol..

[4] Samuel V.T. (2011). Fructose induced lipogenesis: From sugar to fat to insulin resistance. Trends Endocrinol. Metab..

[5] Lim J.S., Mietus-Snyder M., Valente A., Schwarz J.M., Lustig R.H. (2010). The role of fructose in the pathogenesis of NAFLD and the metabolic syndrome. Nat. Rev. Gastroenterol. Hepatol..

[6] Lustig R.H., Mulligan K., Noworolski S.M., Tai V.W., Wen M.J., Erkin-Cakmak A., Gugliucci A., Schwarz J.M. (2016). Isocaloric fructose restriction and metabolic improvement in children with obesity and metabolic syndrome. Obesity.

[7] Moore J.B., Gunn P.J., Fielding B.A. (2014). The role of dietary sugars and de novo lipogenesis in non-alcoholic fatty liver disease. Nutrients.

[8] Mamikutty N., Thent Z.C., Sapri S.R., Sahruddin N.N., Mohd Yusof M.R., Haji Suhaimi F. (2014). The establishment of metabolic syndrome model by induction of fructose drinking water in male wistar rats. Biomed. Res. Int..

[9] Zhang L., Yang B., Yu B. (2015). Paeoniflorin protects against nonalcoholic fatty liver disease induced by a high-fat diet in mice. Biol. Pharm. Bull..

[10] Ma Z., Liu H., Wang W., Guan S., Yi J., Chu L. (2017). Paeoniflorin suppresses lipid accumulation and alleviates insulin resistance by regulating the Rho kinase/IRS-1 pathway in palmitate-induced HepG_2_ cells. Biomed. Pharmacother..

[11] Ma Z., Chu L., Liu H., Li J., Zhang Y., Liu W., Dai J., Yi J., Gao Y. (2016). Paeoniflorin alleviates non-alcoholic steatohepatitis in rats: Involvement with the ROCK/NF-κB pathway. Int. Immunopharmacol..

[12] Li J.M., Li Y.C., Kong L.D., Hu Q.H. (2010). Curcumin inhibits hepatic protein-tyrosine phosphatase 1B and prevents hypertriglyceridemia and hepatic steatosis in fructose-fed rats. Hepatology.

[13] Kalavalapalli S., Bril F., Koelmel J.P., Abdo K., Guingab J., Andrews P., Li W.Y., Jose D., Yost R.A., Frye R.F. (2018). Pioglitazone improves hepatic mitochondrial function in a mouse model of nonalcoholic steatohepatitis. Am. J. Physiol. Endocrinol. Metab..

[14] Shahataa M.G., Mostafa-Hedeab G., Ali E.F., Mahdi E.A., Mahmoud F.A. (2016). Effects of telmisartan and pioglitazone on high fructose induced metabolic syndrome in rats. Can. J. Physiol. Pharmacol..

[15] Folch J., Lees M., Sloane Stanley G.H. (1957). A simple method for the isolation and purification of total lipids from animal tissues. J. Biol. Chem..

[16] Abd El-Haleim E.A., Bahgat A.K., Saleh S. (2016). Resveratrol and fenofibrate ameliorate fructose-induced nonalcoholic steatohepatitis by modulation of genes expression. World J. Gastroenterol..

[17] Kraegen E.W., Clark P.W., Jenkins A.B., Daley E.A., Chisholm D.J., Storlien L.H. (1991). Development of muscle insulin resistance after liver insulin resistance in high-fat-fed rats. Diabetes.

[18] Softic S., Cohen D.E., Kahn C.R. (2016). Role of dietary fructose and hepatic de novo lipogenesis in fatty liver disease. Dig. Dis. Sci..

[19] Lambert J.E., Ramos-Roman M.A., Browning J.D., Parks E.J. (2014). Increased de novo lipogenesis is a distinct characteristic of individuals with nonalcoholic fatty liver disease. Gastroenterology.

[20] Jang C., Hui S., Lu W., Cowan A.J., Morscher R.J., Lee G., Liu W., Tesz G.J., Birnbaum M.J., Rabinowitz J.D. (2018). The small intestine converts dietary fructose into glucose and organic acids. Cell Metab..

[21] Chong M.F., Fielding B.A., Frayn K.N. (2007). Mechanisms for the acute effect of fructose on postprandial lipemia. Am. J. Clin. Nutr..

[22] Horton J.D., Bashmakov Y., Shimomura I., Shimano H. (1998). Regulation of sterol regulatory element binding proteins in livers of fasted and refed mice. Proc. Natl. Acad. Sci. USA.

[23] Miyazaki M., Dobrzyn A., Man W.C., Chu K., Sampath H., Kim H.J., Ntambi J.M. (2004). Stearoyl-CoA desaturase 1 gene expression is necessary for fructose-mediated induction of lipogenic gene expression by sterol regulatory element-binding protein-1c-dependent and -independent mechanisms. J. Biol. Chem..

[24] Lirio L.M., Forechi L., Zanardo T.C., Batista H.M., Meira E.F., Nogueira B.V., Mill J.G., Baldo M.P. (2016). Chronic fructose intake accelerates non-alcoholic fatty liver disease in the presence of essential hypertension. J. Diabetes Complicat..

[25] Chiu S., Sievenpiper J.L., de Souza R.J., Cozma A.I., Mirrahimi A., Carleton A.J., Ha V., Di Buono M., Jenkins A.L., Leiter L.A. (2014). Effect of fructose on markers of non-alcoholic fatty liver disease (NAFLD): A systematic review and meta-analysis of controlled feeding trials. Eur. J. Clin. Nutr..

[26] Kim W.S., Lee Y.S., Cha S.H., Jeong H.W., Choe S.S., Lee M.R., Oh G.T., Park H.S., Lee K.U., Lane M.D. (2009). Berberine improves lipid dysregulation in obesity by controlling central and peripheral AMPK activity. Am. J. Physiol. Endocrinol. Metab..

[27] Lin M.J., Dai W., Scott M.J., Li R., Zhang Y.Q., Yang Y., Chen L.Z., Huang X.S. (2017). Metformin improves nonalcoholic fatty liver disease in obese mice via down-regulation of apolipoprotein A5 as part of the AMPK/LXRα signaling pathway. Oncotarget.

[28] Smith B.K., Marcinko K., Desjardins E.M., Lally J.S., Ford R.J., Steinberg G.R. (2016). Treatment of nonalcoholic fatty liver disease: Role of AMPK. Am. J. Physiol. Endocrinol. Metab..

[29] Buettner R., Bettermann I., Hechtl C., Gabele E., Hellerbrand C., Scholmerich J., Bollheimer L.C. (2010). Dietary folic acid activates AMPK and improves insulin resistance and hepatic inflammation in dietary rodent models of the metabolic syndrome. Horm. Metab. Res..

[30] Hillgartner F.B., Salati L.M., Goodridge A.G. (1995). Physiological and molecular mechanisms involved in nutritional regulation of fatty acid synthesis. Physiol. Rev..

[31] Li Y., Xu S., Mihaylova M.M., Zheng B., Hou X., Jiang B., Park O., Luo Z., Lefai E., Shyy J.Y. (2011). AMPK phosphorylates and inhibits SREBP activity to attenuate hepatic steatosis and atherosclerosis in diet-induced insulin-resistant mice. Cell Metab..

[32] Gugliucci A. (2016). Fructose surges damage hepatic adenosyl-monophosphate-dependent kinase and lead to increased lipogenesis and hepatic insulin resistance. Med. Hypotheses.

[33] Woods A., Johnstone S.R., Dickerson K., Leiper F.C., Fryer L.G., Neumann D., Schlattner U., Wallimann T., Carlson M., Carling D. (2003). LKB1 is the upstream kinase in the AMP-activated protein kinase cascade. Curr. Biol..

[34] Woods A., Dickerson K., Heath R., Hong S.P., Momcilovic M., Johnstone S.R., Carlson M., Carling D. (2005). Ca^2+^/calmodulin-dependent protein kinase kinase-beta acts upstream of AMP-activated protein kinase in mammalian cells. Cell Metab..

[35] Li P., Koike T., Qin B., Kubota M., Kawata Y., Jia Y.J., Oshida Y. (2008). A high-fructose diet impairs Akt and PKCzeta phosphorylation and GLUT4 translocation in rat skeletal muscle. Horm. Metab. Res..

[36] Wu Y.M., Jin R., Yang L., Zhang J., Yang Q., Guo Y.Y., Li X.B., Liu S.B., Luo X.X., Zhao M.G. (2013). Phosphatidylinositol 3 kinase/protein kinase b is responsible for the protection of paeoniflorin upon H_2_O_2_-induced neural progenitor cell injury. Neuroscience.

